# Association Between Subjective Well-being and Living Longer Without Disability or Illness

**DOI:** 10.1001/jamanetworkopen.2019.6870

**Published:** 2019-07-10

**Authors:** Paola Zaninotto, Andrew Steptoe

**Affiliations:** 1 Department of Epidemiology and Public Health, University College London, London, United Kingdom

## Abstract

**Importance:**

Subjective well-being is associated with reduced mortality, but it is not clear whether additional time is spent in good health or with chronic disease and disability.

**Objective:**

To evaluate the associations between affective well-being, total life expectancy, and life expectancy free of disability and chronic disease.

**Design, Setting, and Participants:**

This survey study used data on 9761 participants from the English Longitudinal Study of Ageing who were followed up for a maximum of 10 years (mean [SD] follow-up, 6 [3.7] years). Discrete-time multistate life table models were used to estimate total life expectancy and life expectancy free of disability or chronic disease. Data were collected between March 2002 and March 2013 and analyzed from December 2018 to April 2019. Analyses were adjusted for wealth and cohabiting status.

**Main Outcomes and Measures:**

The main outcome was life expectancy free of disability and chronic disease. Affective well-being was assessed at baseline as a combination of enjoyment of life and the lack of significant depressive symptoms. Disability was measured in terms of impaired activities of daily living and instrumental activities of daily living, and chronic disease as the occurrence of 6 serious illnesses.

**Results:**

Data were analyzed from 9761 participants (5297 [54%] female; mean [SD] age at baseline, 64 [9.9] years). High affective well-being was associated with longer life expectancy and with longer disability-free and chronic disease–free life expectancies. For example, a woman aged 50 years who reported high affective well-being could expect to live 6 years longer than a woman of similar age with low well-being; 31.4 of her remaining years (95% CI, 30.5-31.9 years) would be likely to be free of disability, compared with 20.8 years (95% CI, 20.1-22.1 years) for a woman with low affective well-being. A man aged 50 years with high affective well-being could expect to live 20.8 years (95% CI, 18.7-22.4 years) without chronic disease, compared with 11.4 years (95% CI, 8.5-14.6 years) for a man reporting low well-being. Similar patterns were observed at the ages of 60, 70, and 80 years.

**Conclusions and Relevance:**

This study suggests that people who report high levels of subjective well-being live longer and also healthier lives than those with lower well-being. These findings add weight to endeavors to promote the subjective well-being of older people.

## Key Points

**Question:**

Longitudinal observational studies indicate that greater subjective well-being is associated with longer survival, but are these additional years spent in good health?

**Findings:**

In this survey study of 9761 older men and women from the English Longitudinal Study of Ageing who were followed up for a maximum of 10 years, higher affective well-being was associated not only with longer life expectancy at older ages, but also with a greater proportion of additional years in good health without chronic disease or disability.

**Meaning:**

Subjective well-being is associated with healthier aging as well as greater longevity, but it is not yet known whether programs to enhance well-being will extend healthy life expectancy.

## Teaser

This survey study evaluates the association between subjective well-being and life expectancy free of disability and chronic disease in older adults in England.

## Introduction

Subjective well-being (SWB) has emerged as an important issue in health research and practice over recent decades and includes experiences of affective well-being such as happiness and enjoyment of life, evaluative well-being related to life satisfaction, and eudemonic well-being, involving judgements of life having meaning and purpose.^1^ There is accumulating evidence that greater SWB, particularly affective well-being, is associated with reduced mortality in prospective epidemiological cohort studies^2,3^ after adjustment for initial health status and other confounders. Although there are discrepancies in the literature,^4^ a meta-analysis^5^ of 62 studies published up to 2016 reported a pooled mortality hazard ratio (HR) of 0.920 (95% CI, 0.905-0.934) for individuals reporting high vs lower SWB.

People experiencing greater SWB may live longer, but is this additional time spent in good health? Associations between SWB and reduced incidence of serious diseases have been described,^6,7,8^ but the evidence is inconsistent.^9,10,11^ There is limited evidence about the association between SWB and future disability.^12,13^

A method of quantifying both the quality and duration of life is the computation of health expectancy. The concept of health expectancy was introduced to address the question of whether people are living longer healthy lives as well as longer lives. Thus, health expectancy provides estimates of how many years of future life are likely to be spent in good health and has been applied to issues such as socioeconomic deprivation, lifestyle, and obesity.^14,15,16^ Here, we estimate life expectancy and healthy life expectancy, defined as years free of disability and serious chronic illness, in association with affective well-being. We measured affective well-being as enjoyment of life coupled with low levels of depressive symptoms, as both aspects of well-being have previously been shown to be associated with mortality in the English Longitudinal Study of Ageing (ELSA).^17,18,19^ We hypothesized that individuals reporting high enjoyment and no depression would live longer lives with less disability and chronic disease than those experiencing low enjoyment of life and depressive symptoms. Because of well-established differences in affective well-being between men and women,^20^ and higher life expectancy in women compared with men, we report our results by sex.

## Methods

### Study Participants

We used data from the first 6 waves of ELSA to calculate life and health expectancies from the age of 50 years. The English Longitudinal Study of Ageing is an open access, nationally representative biennial longitudinal survey of those aged 50 years and older living in private households in England.^19^ The sample size was 11 391 people at the first wave in 2002 to 2003 (eFigure in the Supplement). After excluding 158 proxy interviews (people with dementia, Alzheimer disease, and Parkinson disease) and those with nonvalid answers on the measures under consideration, the analytical sample consisted of 9761 individuals (5279 women) in wave 1 (7469 in wave 2, 6291 in wave 3, 5447 in wave 4, 5063 in wave 5, and 4484 in wave 6). The study was approved by the UK National Research Ethics Service, and all participants provided written informed consent. This study followed the Strengthening the Reporting of Observational Studies in Epidemiology (STROBE) reporting guideline.

### Measures

We defined 2 health expectancy outcomes: disability-free life expectancy and chronic disease–free life expectancy using occurrence of chronic conditions.

#### Disability

At each wave, all participants were asked whether they had difficulties in performing activities of daily living (ADL) (dressing, walking across a room, bathing or showering, eating, getting in and out of bed, and using the toilet) and instrumental activities of daily living (IADL) (using a map, preparing a hot meal, shopping for groceries, making phone calls, taking medications, doing housework, and managing money). Responses were summed and categorized as no disability (0 or 1 ADL or IADL) and disability (≥2 ADL or IADL). The cutoff of 2 or more ADL or IADL was chosen based on the average number of ADL or IADL limitations reported by people who at baseline were in receipt of health or disability benefits. Health expectancy based on disability is named here as disability-free life expectancy.

#### Chronic Diseases

Presence of the following chronic health conditions was ascertained at every wave by asking participants whether a physician had ever told them that they have (1) coronary heart disease, (2) stroke, (3) pulmonary disease (chronic bronchitis or emphysema), (4) cancer, (5) diabetes, or (6) arthritis. Conditions not assessed at each wave were not included. Individuals were defined as having a chronic health condition if they reported 1 or more of these conditions. The presence of chronic diseases at baseline (first observation included in analysis) included any chronic conditions reported before the age of 50 years from available information on respondents. Health expectancy based on chronic conditions is named here as chronic disease–free life expectancy.

#### Other Measures

Mortality was ascertained from linked register data with follow-up censored in March 2013.

Enjoyment of life was measured at baseline (2002-2003, wave 1) with 4 items from the CASP-19 quality of life instrument, as described previously.^13,17,19^ Each item (“I enjoy the things that I do”; “I enjoy being in the company of others”; “On balance, I look back on my life with a sense of happiness”; “I feel full of energy these days”) was assessed on a 4-point scale from 0, indicating never, to 3, indicating often, and was subsequently coded as 0, never or rarely and 1, sometimes and often. The 4 binary items were summed to generate the number of items for which enjoyment was reported, with a score ranging from 0 to 4. To interpret the meaning of high enjoyment more specifically, from this score we generated a variable for higher enjoyment defined as 0 indicating no enjoyment if reported fewer than 2 scores and 1 indicating high enjoyment if reported 3 to 4 scores (ie, enjoyment on 3 or 4 of the items).

Depressive symptoms were measured at baseline (2002-2003, wave 1) using the 8-item Center for Epidemiologic Studies Depression Scale.^21^ Participants were asked whether they had experienced each item much of the time during the past week. The scale had good internal consistency at each wave (Cronbach α ≥ 0.95). A score of 4 or more was used to indicate elevated depressive symptoms, which corresponds to the cut point of 16 or more on the full 20-item Center for Epidemiologic Studies Depression Scale.^22^

From responses to the 2 levels of enjoyment of life and the 2 levels of depression, we generated a categorical variable with the following categories: (1) low enjoyment of life and depressive symptoms, (2) high enjoyment and depressive symptoms, (3) low enjoyment and no depression, and (4) high enjoyment and no depression.

Socioeconomic status at baseline was indexed by 3 equal tertiles of total household wealth, including financial wealth (savings and investments), the value of any home and other property (less mortgage), the value of any business assets, and physical wealth, such as artwork and jewelry, net of debt.

From information on marital and cohabiting status, we derived a dichotomous variable for cohabiting with a partner (0, currently cohabiting with a partner whether married or not and 1, currently not cohabiting with a partner).

### Statistical Analysis

Total length of time in study was 10 years (from 2002-2003 to 2012-2013, mean [SD] follow-up, 6 [3.7] years). By the end of follow-up, 2044 deaths occurred. Response rates are reported in eTable 1 in the Supplement and attrition rates in eTable 2 in the Supplement. A comparison of sample characteristics according to the sample size achieved is available in eTable 3 in the Supplement.

A full description of the method used to compute healthy life expectancies is available in the eAppendix in the Supplement. Briefly, we used multistate life table models^23^ suitable for longitudinal data to estimate total life expectancy as well as health expectancies from the ages of 50 to 100 years, separately for the 2 measures of health expectancy: disability free and chronic disease free. We defined the following 3 health states: healthy, unhealthy, and dead. There are separate models for each of the transitions. For disability-free life expectancy there were 4 possible transitions between the health states, namely, healthy to unhealthy (onset), unhealthy to healthy (recovery), healthy to dead, and unhealthy to dead. For chronic disease–free life expectancy, there were only 3 possible transitions as, by definition, recovery was not possible.

We used the Stochastic Population Analysis for Complex Events (SPACE) program^23^ in SAS statistical software version 9.2 (SAS Institute Inc) to estimate multistate life table functions. There are 2 main components to this program: the data component, which prepares the input data sets, and the statistical component, in which transition probabilities and the multistate life table functions and their variances are estimated. Specifically, during the statistical component, age-specific transition probabilities for all possible transitions are estimated from the data using multinomial logistic regression conditional on age, sex, and well-being factors; wealth; and cohabiting with a partner. Health expectancies for ages 50 years and older are then calculated based on these estimated transition probabilities using a stochastic (microsimulation) approach. By using microsimulation, it is possible to simulate the life paths of the members of the population in order to derive several summary statistics of the population dynamics. Each analytical outcome was modeled separately. The program generated individual trajectories for a simulated cohort of 100 000 persons with distributions of covariates at the starting point based on the observed study-specific prevalence by 5-year age group and sex. Analyses were run for the well-being variable as the main exposure and adjusted for wealth and cohabiting with a partner. Variability for these multistate life table estimates (variances, standard errors, and corresponding 95% confidence intervals) were computed using a bootstrap method with 500 replicates for the whole analysis process (multinomial analysis and simulation steps). This method takes account of attrition from the study under the missing-at-random assumption.^24^ We ran a sensitivity analysis to assess the robustness of our findings for the sample of completers.

In eTable 4 in the Supplement, we also present the proportion of remaining life spent without disability and without chronic conditions computed as the ratio of estimates of healthy life expectancy and total life expectancy multiplied by 100.

## Results

Data were analyzed from 9761 participants (5279 [54%] female; mean [SD] age at baseline, 64 [9.9] years). The baseline (2002-2003) characteristics of the sample are presented in Table 1 by 10-year age groups. The prevalence of people reporting high affective well-being (enjoyment of life and no depression) at baseline ranged from 52.9% in the youngest age group to 47.2% in the oldest. The prevalence of disability, defined as difficulties performing basic ADLs and IADLs, increased with age (from 8.9% in those aged 50-59 years to 30.0% in those aged ≥80 years). Older people also had a higher prevalence of chronic disease (69.1% in those aged ≥80 years vs 36.2% in those aged 50-59 years). The prevalence of people in the richest wealth tertile decreased with age, ranging from 37.8% in the youngest age group to 24.2% in those aged 80 years or older; older people were more likely to not be cohabiting with a partner than younger people (64.6% vs 19.8%).

**Table 1.** Sample Characteristics at Baseline, England, 2002 to 2003

Characteristic	Age, %				Total
	50-59 y	60-69 y	70-79 y	≥80 y	
No. (%)	3680 (37.7)	3008 (30.8)	2147 (22.0)	926 (9.5)	9761 (100)
Men	46.1	47.3	46.2	40.2	45.9
Women	53.9	52.7	53.8	59.8	54.1
Affective well-being					
Enjoyment of life and depressive symptoms					
Low	11.7	10.0	10.8	12.1	11.0
High	3.2	3.7	5.5	7.7	4.3
Enjoyment of life and no depression					
Low	32.2	30.8	29.8	33.1	31.3
High	52.9	55.6	53.9	47.2	53.4
Disability	8.9	11.3	15.0	30.0	13.0
Chronic diseases	36.2	51.4	63.4	69.1	50.0
Wealth					
Low	27.9	27.5	36.2	46.4	31.4
Middle	34.2	35.3	34.6	29.4	34.2
High	37.8	37.3	29.2	24.2	34.5
Not cohabiting with a partner	19.8	24.5	38.2	64.6	29.6

Life expectancy, disability-free life expectancy, and life expectancy with disability estimates according to affective well-being are presented in Table 2 for men and Table 3 for women at the ages of 50, 60, 70, and 80 years. Values are adjusted for wealth and cohabiting status. At the age of 50 years, life expectancy for those experiencing low enjoyment and depression was 27.4 years in men and 31.0 years in women (difference, 3.6 years; 95% CI, 2.9-4.3 years; *P* < .001), compared with 33.0 years in men and 36.5 years in women reporting high enjoyment of life and no depression (difference, 3.5 years; 95% CI, 2.6-4.4 years; *P* < .001). Men and women experiencing high enjoyment and no depression could expect to live an additional 29.4 (95% CI, 28.8-30.0 years) and 31.4 years (95% CI, 30.5-31.9 years), respectively (difference, 2.0 years; 95% CI, 0.9-3.1 years; *P* < .001), of their remaining lives free from disability. Disability-free life expectancy at the age of 50 years was 19.7 years (95% CI, 19.3-21.2 years) for men and 20.8 years (95% CI, 20.1-22.1 years) for women who experienced low enjoyment of life and depression. Thus, the estimated years expected to live with disability are greater among men and women who experienced low than high affective well-being. Both components of affective well-being contribute to this pattern. Thus, among women, experiencing depressive symptoms even though enjoyment of life was high was associated with a 2-year reduction in disability-free life expectancy, compared with low enjoyment of life and no depressive symptoms (95% CI, 1.1-3.3 years; *P* < .001). At the age of 60, 70, and 80 years, life expectancy and disability-free life expectancy estimates were lower, and the estimated number of years expected to live with disability, higher. However, a similar pattern to that observed at the age of 50 years emerged, with significant differences between people experiencing low enjoyment of life and depression compared with high enjoyment of life and no depression. For example, men aged 70 years with high enjoyment of life and no depression would be expected to live 12.7 years without disability compared with 4.8 years for those experiencing low enjoyment of life and depressive symptoms; the corresponding estimates for women were 14.2 vs 6.9 years.

**Table 2.** Life Expectancy, Disability-Free Life Expectancy, and Life Expectancy With Disability According to Affective Well-being Among Men, England, 2002 to 2013

Affective Well-being	Life Expectancy, y^a^		
	Total (95% CI)	Disability Free (95% CI)	With Disability (95% CI)
**Age 50 y**			
Enjoyment of life and depressive symptoms			
Low	27.4 (27.0-28.4)	19.7 (19.3-21.2)	7.7 (6.3-8.3)
High	30.7 (28.5-32.2)	24.9 (22.9-26.0)	5.2 (4.6-6.5)
Enjoyment of life and no depression			
Low	30.4 (29.8-31.2)	26.2 (25.7-26.9)	4.2 (3.9-4.5)
High	33.0 (32.5-33.6)	29.4 (28.8-30.0)	3.6 (3.4-3.9)
**Age 60 y**			
Enjoyment of life and depressive symptoms			
Low	18.4 (17.8;19.3)	11.9 (11.5-12.8)	6.6 (5.7-7.2)
High	20.3 (19.3-22.2)	15.0 (13.7-16.4)	5.3 (4.7-6.5)
Enjoyment of life and no depression			
Low	21.7 (21.2-22.3)	17.7 (17.3-18.2)	3.9 (3.6-4.1)
High	24.1 (23.4-24.6)	20.8 (20.2-21.2)	3.3 (3.1-3.5)
**Age 70 y**			
Enjoyment of life and depressive symptoms			
Low	10.9 (5.6-12.0)	4.8 (6.0-6.3)	5.2 (6.4-8.0)
High	12.8 (12.3-14.4)	8.0 (7.5-9.5)	4.8 (4.1-5.4)
Enjoyment of life and no depression			
Low	13.5 (13.1-14.0)	10.3 (10.0-10.8)	3.2 (2.9-3.4)
High	15.6 (15.0-16.1)	12.7 (12.1-13.1)	2.9 (2.7-3.2)
**Age 80 y**			
Enjoyment of life and depressive symptoms			
Low	6.9 (6.2-7.2)	3.2 (2.7-3.7)	3.7 (3.0-4.0)
High	7.5 (7.2-8.4)	5.0 (4.7-5.6)	2.5 (2.2-2.9)
Enjoyment of life and no depression			
Low	7.9 (7.6-8.2)	5.3 (5.0-5.6)	2.6 (2.3-2.8)
High	9.3 (8.5-9.7)	7.1 (6.1-7.3)	2.2 (2.1-2.6)

^a^ Estimates adjusted for wealth and cohabiting status.

**Table 3.** Life Expectancy, Disability-Free Life Expectancy, and Life Expectancy With Disability According to Affective Well-being Among Women, England, 2002 to 2013

Affective Well-being	Life Expectancy, y^a^		
	Total (95% CI)	Disability Free (95% CI)	With Disability (95% CI)
**Age 50 y**			
Enjoyment of life and depressive symptoms			
Low	31.0 (30.4-31.9)	20.8 (20.1-22.1)	10.2 (9.3-10.9)
High	33.7 (32.4-35.3)	25.9 (24.7-27.1)	7.8 (6.9-9.1)
Enjoyment of life and no depression			
Low	33.4 (33.5-39.4)	28.3 (27.7-28.9)	6.1 (5.4-6.4)
High	36.5 (35.8-37.0)	31.4 (30.5-31.9)	5.1 (4.9-5.6)
**Age 60 y**			
Enjoyment of life and depressive symptoms			
Low	22.9 (22.1-23.4)	13.6 (13.2-14.9)	9.3 (8.1-9.4)
High	24.5 (23.5-25.7)	17.3 (16.3-18.4)	7.2 (6.6-8.4)
Enjoyment of life and no depression			
Low	24.9 (24.2-25.4)	19.2 (18.6-19.7)	5.7 (5.2-6.2)
High	27.6 (27.1-28.1)	22.8 (22.2-23.4)	4.8 (4.5-5.0)
**Age 70 y**			
Enjoyment of life and depressive symptoms			
Low	14.6 (14.0-15.2)	6.9 (6.4-7.8)	7.7 (7.0-8.2)
High	15.8 (15.1-17.5)	9.3 (8.3-10.4)	6.5 (5.8-7.6)
Enjoyment of life and no depression			
Low	16.6 (15.8-17.5)	11.6 (11.1-12.3)	5.0 (4.5-5.3)
High	18.6 (18.1-19.1)	14.2 (13.6-14.6)	4.5 (4.1-4.7)
**Age 80 y**			
Enjoyment of life and depressive symptoms			
Low	9.0 (8.4-9.4)	3.4 (3.0-4.0)	5.6 (4.9-5.8)
High	9.7 (9.3-11.0)	5.0 (4.5-5.7)	4.8 (4.3-5.6)
Enjoyment of life and no depression			
Low	10.1 (9.5-10.5)	6.3 (5.9-6.6)	3.8 (3.4-4.1)
High	11.3 (10.7-11.6)	7.7 (7.2-8.0)	3.6 (3.4-3.9)

^a^ Estimates adjusted for wealth and cohabiting status.

The estimates of healthy life expectancy based on chronic health conditions are summarized in Table 4 for men and Table 5 for women. At the age of 50, men and women who experienced low enjoyment of life and depressive symptoms could expect to live an additional 11.4 (95% CI, 8.5-14.6 years) to 11.9 years (95% CI, 9.0-14.7 years) of their lives free from a chronic disease, compared with 20.8 (95% CI, 18.7-22.4) to 22.2 (95% CI, 20.2-24.3) additional years for those who experienced high enjoyment and no depressive symptoms. Men and women who experienced low enjoyment of life and depressive symptoms could expect to live 5 extra years with chronic disease compared with those reporting high affective well-being. There were no differences in the chronic disease–free life expectancy estimates between the 2 middle categories of well-being (high enjoyment with depressive symptoms and low enjoyment with no depressive symptoms). Estimates of chronic disease–free life expectancy were in general lower than estimates of disability-free life expectancy, regardless of affective well-being. Similar differences by affective well-being are observed at the ages of 60, 70, and 80 years. Thus at age 60 years, the difference in chronic disease–free life expectancy was 7 to 8 years for men and women experiencing high enjoyment with no depressive symptoms and low enjoyment with depressive symptoms.

**Table 4.** Life Expectancy, Chronic Disease–Free Life Expectancy, and Life Expectancy With Chronic Disease According to Affective Well-being Among Men, England, 2002 to 2013

Affective Well-being	Life Expectancy, y^**a**^		
	Total (95% CI)	Chronic Disease Free (95% CI)	With Chronic Disease (95% CI)
**Age 50 y**			
Enjoyment of life and depressive symptoms			
Low	30.2 (28.6-31.9)	11.4 (8.5-14.6)	18.8 (15.7-22.1)
High	32.9 (31.1-35.3)	16.9 (12.1-22.2)	16.0 (11.4-21.3)
Enjoyment of life and no depression			
Low	32.5 (31.7-33.6)	16.9 (14.9-19.5)	15.6 (13.8-17.7)
High	35.1 (34.2-35.9)	20.8 (18.7-22.4)	14.3 (12.7-16.0)
**Age 60 y**			
Enjoyment of life and depressive symptoms			
Low	18.3 (17.1-19.7)	3.9 (2.1-5.7)	14.4 (13.0-16.3)
High	19.9 (18.2-21.9)	2.8 (0.8-6.2)	17.2 (13.6-19.9)
Enjoyment of life and no depression			
Low	21.8 (21.0-22.4)	8.2 (6.9-9.6)	13.5 (12.1-14.6)
High	24.2 (23.5-25.1)	11.5 (10.1-12.9)	12.8 (11.4-13.9)
**Age 70 y**			
Enjoyment of life and depressive symptoms			
Low	11.6 (10.8-12.6)	2.1 (1.0-3.6)	9.4 (7.8-11.0)
High	13.4 (11.9-14.7)	1.1 (0.0-3.5)	12.3 (9.5-13.6)
Enjoyment of life and no depression			
Low	13.5 (12.9-14.2)	3.5 (2.7-4.4)	10.0 (8.9-11.0)
High	15.6 (15.1-16.3)	4.9 (3.8-6.1)	10.8 (9.7-11.8)
**Age 80 y**			
Enjoyment of life and depressive symptoms			
Low	6.7 (5.9-7.4)	0.7 (0.0-1.6)	6.0 (4.5-6.9)
High	7.2 (6.2-8.6)	1.0 (0.0-2.5)	6.1 (4.5-8.1)
Enjoyment of life and no depression			
Low	8.0 (7.5-8.4)	2.2 (1.4-2.9)	5.8 (5.1-6.6)
High	9.4 (8.9-10.0)	2.8 (2.0-3.5)	6.6 (5.8-7.6)

^a^ Estimates adjusted for wealth and cohabiting status.

**Table 5.** Life Expectancy, Chronic Disease–Free Life Expectancy, and Life Expectancy With Chronic Disease According to Affective Well-being Among Women, England, 2002 to 2013

Affective Well-being	Life Expectancy, y^a^		
	Total (95% CI)	Chronic Disease Free (95% CI)	With Chronic Disease (95% CI)
**Age 50 y**			
Enjoyment of life and depressive symptoms			
Low	31.7 (30.2-33.1)	11.9 (9.0-14.7)	19.8 (16.8-22.7)
High	35.0 (32.7-36.6)	16.7 (12.2-22.2)	18.3 (13.6-22.3)
Enjoyment of life and no depression			
Low	34.3 (33.2-35.5)	17.8 (15.7-20.5)	16.6 (14.1-18.7)
High	36.7 (35.7-37.5)	22.2 (20.2-24.3)	14.4 (12.5-16.1)
**Age 60 y**			
Enjoyment of life and depressive symptoms			
Low	22.9 (21.6-24.0)	5.2 (3.3-7.0)	17.7 (15.5-19.5)
High	24.2 (22.5-25.9)	5.8 (2.7-9.0)	18.4 (15.1-22.1)
Enjoyment of life and no depression			
Low	25.0 (24.0-25.7)	9.0 (7.3-10.4)	16.0 (14.3-18.0)
High	27.8 (27.1-28.5)	12.5 (10.6-14.0)	15.2 (14.1-16.8)
**Age 70 y**			
Enjoyment of life and depressive symptoms			
Low	14.7 (13.8-15.8)	2.4 (1.3-3.6)	12.3 (11.0-14.0)
High	15.8 (14.5-17.2)	1.9 (0.8-3.4)	13.9 (12.0-15.4)
Enjoyment of life and no depression			
Low	16.8 (16.0-17.4)	4.0 (2.9-4.8)	12.8 (11.6-13.9)
High	18.8 (18.2-19.4)	6.1 (5.1-7.5)	12.7 (11.5-13.8)
**Age 80 y**			
Enjoyment of life and depressive symptoms			
Low	8.8 (7.9-9.4)	1.6 (0.8-2.8)	7.2 (6.1-8.1)
High	9.9 (8.7-10.8)	1.0 (0.4-1.9)	8.9 (7.4-10.1)
Enjoyment of life and no depression			
Low	9.9 (9.5-10.7)	3.3 (2.2-4.2)	6.6 (5.8-8.2)
High	11.3 (10.9-11.9)	3.0 (2.2-4.0)	8.4 (7.3-9.3)

^a^ Estimates adjusted for wealth and cohabiting status.

We also calculated the proportion of remaining life free from disability and chronic health conditions (eTable 4 in the Supplement) at the ages of 50, 60, 70, and 80 years. In addition, we report the odds ratios for all the possible transitions obtained from multinomial logistic regression models and the model fit in eTable 5 and eTable 6 in the Supplement.

In eTables 7, 8, 9, and 10 in the Supplement, we report the results restricted to completers, that is, respondents who were present at all measurement occasions (4440 individuals). Estimates were very similar to those obtained with the available sample, and the overall conclusions remained unchanged.

## Discussion

Our aim was to establish whether greater SWB at older ages is associated not only with longer life, but also with a healthier life. The results support this hypothesis, showing that people experiencing greater enjoyment of life and no depressive symptoms are likely to live more of their remaining years in good health, assessed in terms of freedom from disability or serious chronic health conditions. The differences are substantial. For instance, a woman aged 50 years who enjoyed life might expect to live a further 37 years compared with 31 years for those who did not enjoy life and had depressive symptoms; 31 of these years, or 86% of remaining life, would be lived without disability, compared with 21 years (67% of remaining life) for a woman reporting low enjoyment and depressive symptoms. Interestingly we found that women who reported depressive symptoms and high enjoyment could expect to live 2 fewer years free from disability compared with women who had no depression and low enjoyment. This result suggests that depressive symptoms are associated with a shorter disability-free life expectancy independently of high enjoyment of life. Furthermore, women in our study had longer mean life spans than men, but these additional years of life are mostly spent with disability or a chronic condition, even among those with highest affective well-being.

Healthy life expectancy has not previously been studied in association with SWB. However, the results are compatible with research indicating that greater SWB is associated with reduced mortality in observational population cohorts.^5^ The greater proportion of life spent without disability or serious illness is also consistent with studies of incident disability and functional decline. For example, an analysis of ELSA indicated that greater enjoyment of life was associated with a reduced risk of impaired ADLs, even after demographic factors, baseline health, depression, and health behaviors (smoking, physical activity, and alcohol intake) had been taken into account.^13^ Longitudinal associations between SWB and incident coronary heart disease, arthritis, and frailty have also been documented.^8,25,26^ Our findings that women with depression and high enjoyment of life could expect to live 2 fewer years without disability than women without depression and low enjoyment of life is not surprising given that women report consistently higher levels of depression than men even at older ages and highlight the association of mental health with life expectancy independent of SWB.^20^

An advantage of the health expectancy approach as opposed to conventional survival analysis is that it provides estimates of the years that might be gained by people experiencing greater SWB. The observation that these added years are likely to be spent in good health allays the concern that greater longevity might come at the expense of health-related quality of life. It adds weight to taking well-being seriously in the health context. The finding that greater SWB is not associated with an increase in number of years spent with disability or serious chronic illness suggests that positive psychological states may be associated with a compression of morbidity.^27^

We analyzed health expectancies using discrete multistate life table models applied to longitudinal data. The multistate life table method has several advantages: it is based on incidence measures representing current health transitions; it allows movement in both directions between all surviving health states; and it allows death rates to differ by health state, therefore taking into account the different mortality profiles by health status. Our findings are generalizable to the general English population of people aged 50 years and older.

Our modeling was based on the observation that enjoyment of life (a positive state) and depressive symptoms are not simply opposite ends of a continuum, and that the two experiences can coexist. As shown in Table 1, a substantial proportion of the study population reported low enjoyment of life without marked depressive symptoms, while a smaller number reported high enjoyment coupled with depressive symptoms. A number of previous studies have documented associations between positive states of SWB and favorable health outcomes after controlling statistically for depressive symptoms, further supporting the partial independence of these experiences.^3^ A person might be in an affectively neutral state, not experiencing distress but not feeling particularly happy either. Additionally, moods fluctuate rapidly, and many experiences are bittersweet, eliciting both positive and negative feelings.^28^ Nevertheless, the largest differences in healthy life expectancy were found between the extreme groups of high enjoyment of life without depressive symptoms and low enjoyment of life combined with depression.

### Limitations

Some limitations should be acknowledged. The associations between SWB and healthy life expectancy reported in this analysis are based on observational data, so they do not imply causality. The associations may result from the effects of unobserved confounding factors. The multistate life tables method does not reduce the problem of unobserved heterogeneity and is robust under the missing-at-random assumption. In sensitivity analysis restricting the data to the sample of completers, we showed that the results were similar and did not change the overall conclusions. Affective well-being and covariates (wealth and cohabiting status) were assessed at baseline, and changes over time in these measures were not considered. Thus, our results should be interpreted under the assumption that these characteristics remain the same. When we further explored this issue, we found few changes over time in depression and well-being or wealth but slightly more variation in cohabiting status. Another possible limitation of our study is that we assessed only a limited number of serious health problems in our index of chronic conditions, and other medical issues might result in different associations.

Nevertheless, there are 2 broad sets of mechanisms that might contribute to the association of SWB with healthy life expectancy. First, greater SWB has been linked with favorable lifestyle choices, including more physical activity, less smoking, better sleep, and more reliable use of preventive health services.^29,30,31^ Healthier lifestyles may help postpone the onset of disability, as well as reduce the risk of chronic physical ill health. Second, SWB is associated with a range of biological processes, including reduced cortisol output, lower concentration of inflammatory cytokines, and higher serum antioxidant levels.^32,33,34^ These processes, in turn, protect against increased disability and risk of coronary heart disease, diabetes, and other serious conditions.^35,36^

Another limitation of our study is the use of self-reported conditions. Objective measures of health conditions would have been preferable. The wording of the questions that respondents are asked to report chronic conditions has been formulated to reduce subjectivity (“Has a doctor ever told you that you have …”). Comparisons of self-reports of chronic conditions with medical records have found acceptable levels of agreement.^37^

## Conclusions

This analysis suggests that people who experience greater affective well-being may live longer healthy lives as well as longer lives. Improving SWB at older ages may have the potential to increase the number of years that older individuals can expect to live in good health. The postponement of disability or chronic illness could, in turn, have implications for expenditure on health care, as fewer people at older ages would make demands on hospital and primary care services.

## Notes

Supplement. **eTable 1.** ELSA Achieved Interview Sample Counts for Core Members and Study Response Rates **eFigure. **Achieved Sample Size at Baseline (2002-2003) **eTable 2.** Sample Size and Attrition Rates by Wave of Assessment, England, 2002-2013 **eTable 3.** Comparisons of Baseline Characteristics Comparing the Completers With Those Who Died and Dropped Out, England, 2002-2003 **eTable 4.** Proportion of Remaining Life Spent Without Disability and Without Chronic Diseases According to Affective Wellbeing, England 2002-2013 **eTable 5.** Odds Ratios for Disability Transition From Multinomial Logistic Models, England, 2002-2013 **eTable 6.** Odds Ratios for Chronic Disease Transition From Multinomial Logistic Models, England, 2002-2013 **eTable 7.** Life Expectancy, Disability-Free Life Expectancy and Life Expectancy With Disability According to Affective Wellbeing Among Men, Complete Sample, England 2002-2013 **eTable 8.** Life Expectancy, Disability-Free Life Expectancy and Life Expectancy With Disability According to Affective Wellbeing Among Women, Complete Sample, England 2002-2013 **eTable 9.** Life Expectancy, Chronic Disease-Free Life Expectancy and Life Expectancy With Chronic Disease According to Affective Wellbeing Among Men, Complete Sample, England 2002-2013 **eTable 10.** Life Expectancy, Chronic Disease-Free Life Expectancy and Life Expectancy With Chronic Disease According to Affective Wellbeing Among Women, Complete Sample, England 2002-2013 **eAppendix.** Computation of Healthy Life Expectancy **eReferences**
