# Supplementary material for: Association Between Subjective Well-being and Living Longer Without Disability or Illness
Source: JAMA Netw Open. 2019 Jul 10;2(7):e196870. doi: 10.1001/jamanetworkopen.2019.6870 (PMC6624816; doi:10.1001/jamanetworkopen.2019.6870)
Supplement: Supplement. — eTable 1. ELSA Achieved Interview Sample Counts for Core Members and Study Response Rates eFigure. Achieved Sample Size at Baseline (2002-2003) eTable 2. Sample Size and Attrition Rates by Wave of Assessment, England, 2002-2013 eTable 3. Comparisons of Baseline Characteristics Comparing the Completers With Those Who Died and Dropped Out, England, 2002-2003 eTable 4. Proportion of Remaining Life Spent Without Disability and Without Chronic Diseases According to Affective Wellbeing, England 2002-2013 eTable 5. Odds Ratios for Disability Transition From Multinomial Logistic Models, England, 2002-2013 eTable 6. Odds Ratios for Chronic Disease Transition From Multinomial Logistic Models, England, 2002-2013 eTable 7. Life Expectancy, Disability-Free Life Expectancy and Life Expectancy With Disability According to Affective Wellbeing Among Men, Complete Sample, England 2002-2013 eTable 8. Life Expectancy, Disability-Free Life Expectancy and Life Expectancy With Disability According to Affective Wellbeing Among Women, Complete Sample, England 2002-2013 eTable 9. Life Expectancy, Chronic Disease-Free Life Expectancy and Life Expectancy With Chronic Disease According to Affective Wellbeing Among Men, Complete Sample, England 2002-2013 eTable 10. Life Expectancy, Chronic Disease-Free Life Expectancy and Life Expectancy With Chronic Disease According to Affective Wellbeing Among Women, Complete Sample, England 2002-2013 eAppendix. Computation of Healthy Life Expectancy eReferences [file jamanetwopen-2-e196870-s001.pdf]

Supplementary Online Content

Zaninotto P, Steptoe A. Association between subjective well-being and living longer without disability or illness. *JAMA Netw Open*. 2019;2(7):e196870. doi:10.1001/jamanetworkopen.2019.6870

eTable 1. ELSA Achieved Interview Sample Counts for Core Members and Study Response Rates
eFigure. Achieved Sample Size at Baseline (2002-2003)
eTable 2. Sample Size and Attrition Rates by Wave of Assessment, England, 2002-2013
eTable 3. Comparisons of Baseline Characteristics Comparing the Completers With Those Who Died and Dropped Out, England, 2002-2003
eTable 4. Proportion of Remaining Life Spent Without Disability and Without Chronic Diseases According to Affective Wellbeing, England 2002-2013
eTable 5. Odds Ratios for Disability Transition From Multinomial Logistic Models, England, 2002-2013
eTable 6. Odds Ratios for Chronic Disease Transition From Multinomial Logistic Models, England, 2002-2013
eTable 7. Life Expectancy, Disability-Free Life Expectancy and Life Expectancy With Disability According to Affective Wellbeing Among Men, Complete Sample, England 2002-2013
eTable 8. Life Expectancy, Disability-Free Life Expectancy and Life Expectancy With Disability According to Affective Wellbeing Among Women, Complete Sample, England 2002-2013
eTable 9. Life Expectancy, Chronic Disease-Free Life Expectancy and Life Expectancy With Chronic Disease According to Affective Wellbeing Among Men, Complete Sample, England 2002-2013
eTable 10. Life Expectancy, Chronic Disease-Free Life Expectancy and Life Expectancy With Chronic Disease According to Affective Wellbeing Among Women, Complete Sample, England 2002-2013
eAppendix. Computation of Healthy Life Expectancy
eReferences

This supplementary material has been provided by the authors to give readers additional information about their work.

© 2019 Zaninotto P et al. *JAMA Network Open.*

**eTable 1 ELSA Achieved Interview Sample Counts for Core Members and Study Response Rates***						
	**Wave 1**	**Wave 2**	**Wave3**	**Wave4**	**Wave5**	**Wave6**
	**(2002-2003)**	**(2004-2005)**	**(2006-2007)**	**(2008-2009)**	**(2010-2011)**	**(2012-2013)**
Interviewed	11,391	8,780	7,535	6,623	6,242	5,659
Response rate	67%	82%	73%	74%	69%	66%

*The ‘study response rate’ at a given wave is the proportion of the remaining eligible cohort interviewed at that wave.

© 2019 Zaninotto P et al. *JAMA Network Open.*

eFigure Achieved sample size at baseline (2002-2003)

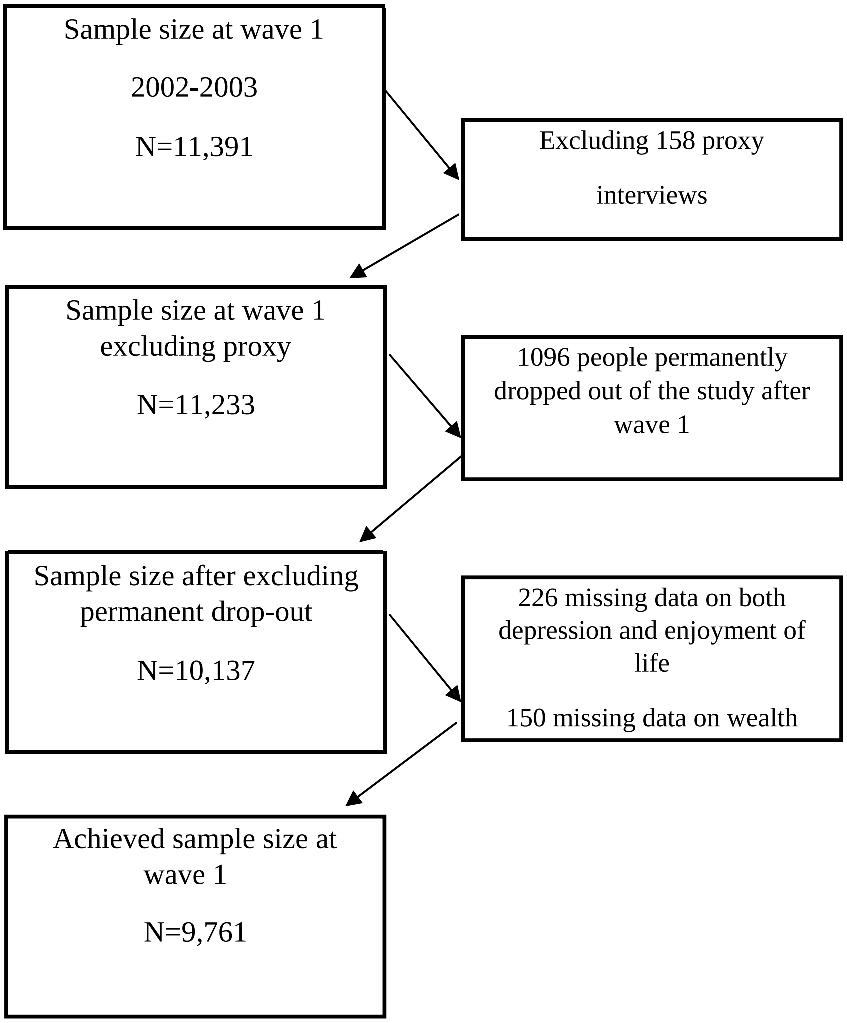

© 2019 Zaninotto P et al. *JAMA Network Open.*

**eTable 2 Sample size and attrition rates by wave of assessment England, 2002-2013**						
	**Wave 1**	**Wave 2**	**Wave3**	**Wave4**	**Wave5**	**Wave6**
	**(2002-2003)**	**(2004-2005)**	**(2006-2007)**	**(2008-2009)**	**(2010-2011)**	**(2012-2013)**
Interviewed	9,761	7,469	6,291	5,447	5,063	4,484
Dropped out*		2,107	2,935	3,411	3,356	3,278
Died*		185	535	903	1342	1966
% Attrition§ due to drop-out		21.6%	30.1%	34.9%	34.4%	33.6%

*Cumulative drop out and deaths (note that 78 deaths occurred after wave 6).
§Attrition is defined as the prevalence of cases that dropped out at each wave (excluding deaths)

eTable 3 Comparisons of baseline characteristics comparing the completers with those who died and dropped out, England, 2002-2003

Characteristics	**Completers**	**Dropped-out**	**Died**
N (%)	4440 (45.5)	3277 (33.6)	2044 (20.9)
Mean age	62	62	74
Men (%)	44.59	43.21	53.13
Women (%)	55.41	56.79	46.87
Affective wellbeing			
Low enjoyment of life and depressive symptoms (%)	9.7	10.4	14.8
High enjoyment of life and depressive symptoms (%)	3.6	3.8	6.3
Low enjoyment of life and no depression (%)	29.8	31.2	34.7
High enjoyment of life and no depression (%)	56.9	54.6	44.1
Disability (%)	9.7	9.5	25.6
Chronic diseases (%)	51.2	37.1	68.1
Low wealth (%)	24.8	31.1	45.9
Middle wealth (%)	34.7	35.8	30.4
High wealth (%)	40.5	33.1	23.7
Not cohabiting with a partner (%)	26.4	24.8	43.9

© 2019 Zaninotto P et al. *JAMA Network Open.*

eTable 4 Proportion of Remaining Life Spent Without Disability and Without Chronic Diseases according to Affective Wellbeing, England 2002-2013

Affective wellbeing	**Proportion without disability* %**	**(95% CI)**	**Proportion without chronic diseases* %**	**(95% CI)**
**Age 50 men**				
Low enjoyment of life and depressive symptoms	71.9	(70.2; 77.1)	40.6	(29.7; 49.7)
High enjoyment of life and depressive symptoms	82.8	(79.2; 84.5)	49.7	(33.7; 62.7)
Low enjoyment of life no depression	86.1	(85.3; 87.1)	51.8	(46.3; 56.9)
High enjoyment of life no depression	89.1	(88.4; 89.7)	58.8	(52.9; 63.4)
**Age 60 men**				
Low enjoyment of life and depressive symptoms	64.2	(62.3; 68.4)	21.3	(11.8; 29.4)
High enjoyment of life and depressive symptoms	73.8	(70.1; 77.4)	13.8	(4.2; 30.3)
Low enjoyment of life no depression	81.9	(81.0; 83.3)	37.9	(32.7; 43.9)
High enjoyment of life no depression	86.5	(85.5; 87.2)	47.4	(42.2; 53.1)
**Age 70 men**				
Low enjoyment of life and depressive symptoms	48.1	(44.6; 54.1)	18.5	(8.6; 31.4)
High enjoyment of life and depressive symptoms	62.3	(59.7; 69.0)	8.5	(0.2; 26.0)
Low enjoyment of life no depression	76.4	(75.1; 78.2)	25.6	(20.3; 32.9)
High enjoyment of life no depression	81.4	(79.9; 82.5)	31.3	(24.8; 37.9)
**Age 80 men**				
Low enjoyment of life and depressive symptoms	46.4	(41.8; 53.3)	10.5	(0.0; 26.2)
High enjoyment of life and depressive symptoms	66.7	(63.8; 71.7)	14.4	(0.0; 34.3)
Low enjoyment of life no depression	67.2	(64.9; 70.0)	27.5	(18.5; 35.8)
High enjoyment of life no depression	75.9	(71.4; 77.6)	30.0	(20.6; 37.2)
**Age 50 women**				
Low enjoyment of life and depressive symptoms	67.2	(65.0; 70.0)	36.2	(29.5; 44.6)
High enjoyment of life and depressive symptoms	76.9	(74.2; 79.5)	45.6	(31.4; 57.7)
Low enjoyment of life no depression	82.4	(81.4; 84.1)	49.3	(43.5; 55.8)
High enjoyment of life no depression	86.0	(85.0; 86.7)	57.3	(51.3; 62.3)
**Age 60 women**				
Low enjoyment of life and depressive symptoms	59.6	(58.6; 63.8)	22.7	(14.5; 30.3)
High enjoyment of life and depressive symptoms	70.7	(67.1; 73.2)	24.1	(11.3; 36.9)
Low enjoyment of life no depression	77.2	(75.4; 78.6)	35.9	(29.5; 42.5)

* Estimates adjusted for wealth and cohabiting status

© 2019 Zaninotto P et al. *JAMA Network Open.*

eTable 5 Odds ratios for Disability Transition from Multinomial Logistic Models, England, 2002-2013

	Transition			
	**Healthy to unhealthy**	**Healthy to death**	**Unhealthy to healthy**	**Unhealthy to death**
Affective wellbeing	OR* (95%CI)	OR* (95%CI)	OR* (95%CI)	OR* (95%CI)
Low enjoyment of life and depressive symptoms	2.5 (2.2; 2.9)	1.7 (1.3; 2.1)	1.5 (1.3; 1.8)	1.8 (1.4: 2.3)
High enjoyment of life and depressive symptoms	1.9 (1.6; 2.3)	1.4 (1.0; 1.8)	1.1 (0.9; 1.4)	1.3 (0.9; 1.9)
Low enjoyment of life no depression	1.4 (1.2; 1.5)	1.3 (1.1; 1.5)	1.1 (0.9; 1.2)	1.2 (1.0; 1.6)
High enjoyment of life no depression	1	1	1	1
**Model fit statistics**				
AIC	25675.445			
SC	25836.692			
-2 Log L	25639.445			

* Estimates adjusted for wealth and cohabiting status

© 2019 Zaninotto P et al. *JAMA Network Open.*

eTable 6 Odds ratios for Chronic Disease Transition from Multinomial Logistic Models, England, 2002-2013

	Transition		
	**Healthy to unhealthy**	**Healthy to death**	**Unhealthy to death**
Affective wellbeing	OR* (95%CI)	OR* (95%CI)	OR* (95%CI)
Low enjoyment of life and depressive symptoms	1.3 (1.0; 1.7)	1.7 (1.2; 2.4)	1.6 (1.3; 1.9)
High enjoyment of life and depressive symptoms	1.4 (0.9; 2.2)	1.8 (1.1; 2.9)	1.3 (1.0; 1.7)
Low enjoyment of life no depression	1.2 (1.0; 1.4)	1.3 (1.1; 1.6)	1.3 (1.1; 1.5)
High enjoyment of life no depression	1	1	1
**Model fit statistics**			
AIC	11930.753		
SC	11947.335		
-2 Log L	11926.753		

* Estimates adjusted for wealth and cohabiting status

© 2019 Zaninotto P et al. *JAMA Network Open.*

eTable 7 Life Expectancy, Disability-free Life Expectancy and Life Expectancy with Disability according to Affective Wellbeing among men complete sample, England 2002-2013

Affective wellbeing	**Total LE***	**95%CI**	**Disability-free LE***	**95%CI**	**LE with disability***	**95%CI**
**Age 50 men**						
Low enjoyment of life and depressive symptoms	26.1	(24.5; 27.1)	19.2	(16.9; 20.4)	6.9	(5.5; 8.6)
High enjoyment of life and depressive symptoms	28.2	(26.4; 31.3)	23.4	(20.9; 26.2)	4.8	(3.7; 5.7)
Low enjoyment of life no depression	29.4	(28.4; 30.1)	25.5	(24.3; 26.3)	3.9	(3.5; 4.5)
High enjoyment of life no depression	31.9	(30.9; 32.9)	28.6	(27.6; 29.6)	3.3	(3.1; 3.6)
**Age 60 men**						
Low enjoyment of life and depressive symptoms	17.2	(15.7; 18.0)	11.2	(9.7; 12.3)	6.0	(5.2; 7.0)
High enjoyment of life and depressive symptoms	19.3	(17.1; 20.8)	14.2	(11.7; 16.0)	5.1	(4.2; 6.0)
Low enjoyment of life no depression	20.8	(19.8; 22.0)	16.9	(15.6; 17.9)	3.9	(3.2; 4.4)
High enjoyment of life no depression	23.1	(22.6; 24.1)	20.0	(19.4; 21.1)	3.1	(2.7; 3.5)
**Age 70 men**						
Low enjoyment of life and depressive symptoms	10.1	(9.3; 10.6)	4.5	(3.4; 5.5)	5.7	(4.8; 6.7)
High enjoyment of life and depressive symptoms	12.6	(11.3; 13.4)	7.9	(6.6; 8.5)	4.7	(3.6; 5.9)
Low enjoyment of life no depression	12.8	(12.1; 13.3)	9.8	(9.2; 10.4)	3.0	(2.7; 3.3)
High enjoyment of life no depression	15.0	(14.6; 15.6)	12.3	(11.8; 12.9)	2.7	(2.5; 3.2)
**Age 80 men**						
Low enjoyment of life and depressive symptoms	6.3	(5.6; 6.9)	3.1	(2.6; 3.9)	3.2	(2.4; 3.7)
High enjoyment of life and depressive symptoms	7.2	(6.6; 8.1)	5.0	(4.4; 5.6)	2.2	(1.7; 2.6)
Low enjoyment of life no depression	7.5	(6.9; 8.0)	5.1	(4.5; 5.4)	2.5	(2.1; 2.8)
High enjoyment of life no depression	8.9	(8.5; 9.3)	6.8	(6.5; 7.2)	2.1	(1.9; 2.2)

* Estimates adjusted for wealth and cohabiting status

© 2019 Zaninotto P et al. *JAMA Network Open.*

eTable 8 Life Expectancy, Disability-free Life Expectancy and Life Expectancy with Disability according to Affective Wellbeing among women complete sample, England 2002-2013

Affective wellbeing	**Total LE***	**95%CI**	**Disability-free LE***	**95%CI**	**LE with disability***	**95%CI**
**Age 50 women**						
Low enjoyment of life and depressive symptoms	29.5	(28.2; 30.7)	20.2	(19.2; 21.1)	9.3	(8.1; 10.0)
High enjoyment of life and depressive symptoms	31.7	(30.4; 34.4)	24.9	(23.4; 27.1)	6.8	(6.2; 8.2)
Low enjoyment of life no depression	33.3	(32.5; 34.2)	27.5	(26.5; 28.7)	5.8	(4.9; 6.9)
High enjoyment of life no depression	35.5	(34.8; 36.2)	30.6	(30.2; 31.3)	4.9	(4.5; 5.3)
**Age 60 women**						
Low enjoyment of life and depressive symptoms	21.4	(19.6; 22.9)	12.9	(11.9; 14.2)	8.5	(7.6; 9.5)
High enjoyment of life and depressive symptoms	23.4	(21.6; 25.8)	16.4	(14.7; 19.7)	7.0	(5.8; 8.2)
Low enjoyment of life no depression	23.6	(23.0; 24.1)	18.1	(17.0; 18.9)	5.6	(4.8; 6.7)
High enjoyment of life no depression	26.6	(25.6; 27.2)	22.0	(21.2; 22.6)	4.6	(4.4; 5.0)
**Age 70 women**						
Low enjoyment of life and depressive symptoms	13.5	(12.2; 14.6)	6.7	(5.7; 7.5)	6.8	(6.1; 7.2)
High enjoyment of life and depressive symptoms	14.8	(13.7; 16.2)	8.7	(7.8; 9.9)	6.0	(5.0; 7.1)
Low enjoyment of life no depression	15.6	(15.1; 16.6)	10.7	(10.2; 11.4)	4.9	(4.5; 5.3)
High enjoyment of life no depression	17.7	(17.2; 18.4)	13.3	(12.9; 14.2)	4.3	(4.1; 4.5)
**Age 80 women**						
Low enjoyment of life and depressive symptoms	8.3	(7.7; 8.9)	3.2	(2.5; 3.9)	5.1	(4.5; 5.4)
High enjoyment of life and depressive symptoms	9.4	(8.4; 10.1)	4.9	(4.2; 5.9)	4.5	(3.8; 5.1)
Low enjoyment of life no depression	9.6	(9.2; 10.1)	5.9	(5.6; 6.4)	3.7	(3.2; 4.3)
High enjoyment of life no depression	10.7	(10.3; 11.0)	7.2	(6.8; 7.5)	3.5	(3.1; 3.9)

* Estimates adjusted for wealth and cohabiting status

© 2019 Zaninotto P et al. *JAMA Network Open.*

eTable 9 Life Expectancy, Chronic disease-free Life Expectancy and Life Expectancy with Chronic disease according to Affective Wellbeing among men complete sample, England 2002-2013

Affective wellbeing	**Total LE***	**95%CI**	**Chronic disease-free LE***	**95%CI**	**LE with Chronic disease ***	**95%CI**
**Age 50 men**						
Low enjoyment of life and depressive symptoms	26.0	(24.9; 27.7)	11.4	(10.0; 14.3)	14.7	(10.7; 16.3)
High enjoyment of life and depressive symptoms	28.5	(27.0; 31.3)	14.0	(6.7; 18.8)	14.5	(11.8; 22.2)
Low enjoyment of life no depression	29.6	(28.7; 30.5)	15.5	(12.9; 17.5)	14.1	(13.0; 16.5)
High enjoyment of life no depression	32.3	(31.2; 33.0)	18.7	(16.9; 20.4)	13.6	(12.2; 15.2)
**Age 60 men**						
Low enjoyment of life and depressive symptoms	17.4	(15.9; 18.6)	3.7	(2.2; 4.1)	13.7	(12.5; 15.3)
High enjoyment of life and depressive symptoms	18.9	(18.3; 20.6)	1.9	(0.0; 5.5)	17.0	(14.0; 20.0)
Low enjoyment of life no depression	20.8	(20.2; 21.3)	7.1	(5.7; 8.3)	13.7	(12.6; 15.2)
High enjoyment of life no depression	23.2	(22.4; 24.2)	10.2	(8.7; 11.8)	13.0	(12.3; 14.2)
**Age 70 men**						
Low enjoyment of life and depressive symptoms	10.8	(9.4; 11.8)	1.5	(0.6; 2.4)	9.3	(7.8; 10.5)
High enjoyment of life and depressive symptoms	12.8	(11.8; 13.8)	1.5	(0.0; 3.0)	11.3	(9.8; 13.8)
Low enjoyment of life no depression	12.7	(12.1; 13.3)	2.9	(1.9; 3.7)	9.9	(9.5; 11.3)
High enjoyment of life no depression	15.1	(14.5; 15.6)	5.0	(4.3; 6.0)	10.0	(9.4; 10.7)
**Age 80 men**						
Low enjoyment of life and depressive symptoms	6.4	(5.3; 6.6)	0.8	(0.3; 1.3)	5.6	(4.4; 6.1)
High enjoyment of life and depressive symptoms	6.9	(6.3; 8.0)	1.1	(0.0; 2.6)	5.8	(4.4; 7.6)
Low enjoyment of life no depression	7.6	(7.2; 7.9)	2.0	(1.6; 2.6)	5.6	(5.0; 6.0)
High enjoyment of life no depression	9.0	(8.3; 9.4)	2.6	(2.2; 3.6)	6.4	(5.3; 7.2)

* Estimates adjusted for wealth and cohabiting status

© 2019 Zaninotto P et al. *JAMA Network Open.*

eTable 10 Life Expectancy, Chronic disease-free Life Expectancy and Life Expectancy with Chronic disease according to Affective Wellbeing among women complete sample, England 2002-2013

Affective wellbeing	**Total LE***	**95%CI**	**Chronic disease-free LE***	**95%CI**	**LE with Chronic disease ***	**95%CI**
**Age 50 women**						
Low enjoyment of life and depressive symptoms	29.7	(27.9; 30.6)	9.8	(6.4; 12.8)	19.9	(15.0; 23.3)
High enjoyment of life and depressive symptoms	31.8	(29.5; 33.1)	13.8	(10.9; 17.4)	18.0	(14.9; 20.4)
Low enjoyment of life no depression	33.1	(32.3; 34.0)	15.3	(11.7; 17.6)	17.8	(15.3; 20.8)
High enjoyment of life no depression	35.5	(34.8; 36.2)	19.5	(17.1; 21.3)	16.0	(13.8; 18.7)
**Age 60 women**						
Low enjoyment of life and depressive symptoms	21.7	(20.6; 23.0)	2.9	(1.7; 4.5)	18.7	(16.7; 20.6)
High enjoyment of life and depressive symptoms	22.9	(22.7; 25.7)	4.7	(1.3; 6.2)	18.2	(17.6; 24.0)
Low enjoyment of life no depression	23.7	(23.0; 24.5)	6.6	(5.0; 7.9)	17.1	(15.7; 18.5)
High enjoyment of life no depression	26.6	(25.8; 27.5)	10.6	(8.7; 12.0)	15.9	(13.8; 18.5)
**Age 70 women**						
Low enjoyment of life and depressive symptoms	13.5	(12.5; 14.3)	2.1	(1.2; 2.7)	11.3	(10.3; 12.5)
High enjoyment of life and depressive symptoms	15.2	(14.5; 16.6)	0.7	(0.0; 2.6)	14.5	(12.3; 15.6)
Low enjoyment of life no depression	15.8	(15.2; 16.4)	2.6	(1.6; 3.3)	13.2	(12.2; 14.1)
High enjoyment of life no depression	18.0	(17.2; 18.6)	4.7	(3.9; 5.9)	13.3	(12.6; 14.4)
**Age 80 women**						
Low enjoyment of life and depressive symptoms	8.1	(7.3; 8.6)	1.1	(0.5; 2.0)	6.9	(5.6; 7.8)
High enjoyment of life and depressive symptoms	9.5	(9.2; 10.7)	1.2	(0.3; 2.3)	8.3	(7.1; 9.8)
Low enjoyment of life no depression	9.6	(9.1; 10.1)	3.0	(2.1; 3.6)	6.6	(5.5; 7.7)
High enjoyment of life no depression	10.8	(10.3; 11.3)	2.9	(2.3; 3.6)	7.9	(6.8; 8.8)

* Estimates adjusted for wealth and cohabiting status

© 2019 Zaninotto P et al. *JAMA Network Open.*

eAppendix. Computation of healthy life expectancy

The computation of health expectancy using the Sullivan method is usually applied to cross-sectional data, and requires life tables and information on age specific proportions of the population in healthy or unhealthy stages. These proportions are prevalence measures of the actual and current health status of a real population and are used to divide years lived in the life table population. With panel data reliable estimates of life table inputs cannot be obtained. The multi-state life table (MSLT) model has been developed to analyse stochastic processes that involve multiple and recurrent events (typical of longitudinal data), in order to estimate expected duration in various states. MSLT method uses a set of transition schedules from healthy, unhealthy and to death estimated using longitudinal data.

The possible transitions among the health states for disability are represented in the following chart

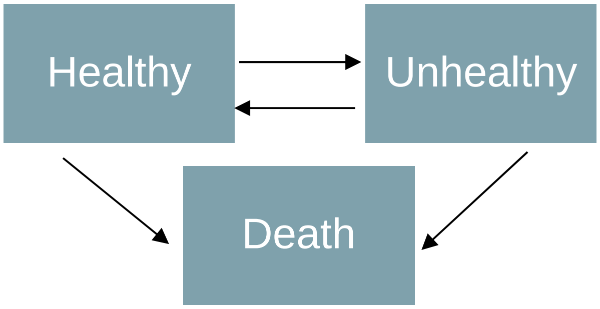

And the transitions among health states for chronic condition in the following, note that recovery is not allowed (from unhealthy to healthy).

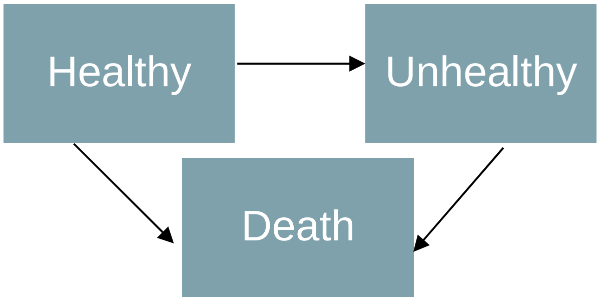

The advantages of multistate life table method are: it is based on incidence measures representing current health transitions; it allows movement in both directions between all surviving health states; it allows death rates to differ by health state so it takes into account the different mortality profiles by health status. The estimation of transition schedules is very important and can be done using logistic regression, multinomial logistic regression or hazard regression.

© 2019 Zaninotto P et al. *JAMA Network Open.*

We used the Stochastic Population Analysis for Complex Events (SPACE)[1] program in SAS 9.2 to estimate MSLT functions. There are two main components to this program: the data component which prepares the input datasets and the statistical component in which transition probabilities and the MSLT functions and their variances are estimated. Specifically, during the data component age-specific transition probabilities for all possible transitions are estimated from the data using multinomial logistic regression conditional on age, sex, well-being factors, wealth and living with a partner. The package also allows the use of survival analysis, however, given that for disability and chronic conditions we do not have exact dates of diagnosis, while we have exact dates of mortality, this would produce and imbalance, therefore multinomial logistic regression is recommended[1]. Health expectancies from the age of 50 and over are then calculated based on these estimated transition probabilities using a stochastic (micro-simulation) approach. By using micro-simulation it is possible to simulate the life paths of the members of the population in order to derive several summary statistics of the population dynamics (an example is available on page 136 of reference 4). The program generates individual trajectories for a simulated cohort of 100,000 persons with distributions of covariates at the starting point based on the observed study-specific prevalence by five year age group and sex. Variability for these MSLT estimates (variances, standard errors and corresponding confidence intervals) are computed using a bootstrap method with 500 replicates for the whole analysis process (multinomial analysis and simulation steps). This method takes account of attrition from the study under the missing at random assumption[1]. More information can be found at http://www.cdc.gov/nchs/data_access/space.htm.

The SPACE manual is available at ftp://ftp.cdc.gov/pub/Health_Statistics/NCHS/Software/space/SPACE_manual.pdf

The annotated codes can be found at

ftp://ftp.cdc.gov/pub/Health_Statistics/NCHS/Software/space/sas/MSLT_RAD2COV_S.pdf

ftp://ftp.cdc.gov/pub/Health_Statistics/NCHS/Software/space/sas/MSLT_SIMxCOV_S.pdf

ftp://ftp.cdc.gov/pub/Health_Statistics/NCHS/Software/space/sas/MSLT_SIMxCOV_M_DX.pdf

ftp://ftp.cdc.gov/pub/Health_Statistics/NCHS/Software/space/sas/SMPEM_SIMDUR.pdf

ftp://ftp.cdc.gov/pub/Health_Statistics/NCHS/Software/space/sas/SMPEM_SIMxCOV_S.pdf

eReferences

1. Cai L, Hayward MD, Saito Y, Lubitz J, Hagedorn A, Crimmins E. Estimation of multi-state life table functions and their variability from complex survey data using the SPACE Program. *Demogr Res.* 2010;22(6):129-158.

© 2019 Zaninotto P et al. *JAMA Network Open.*
